# Editorial comment to “Recurrent episodes of atrioventricular nodal reentrant tachycardia: Sites of ablation success, ablation endpoint, and primary culprits for recurrence”

**DOI:** 10.1002/joa3.13101

**Published:** 2024-06-21

**Authors:** Shiro Nakahara, Yuichi Hori

**Affiliations:** ^1^ Department of Cardiology Dokkyo Medical University Saitama Medical Center Koshigaya Japan

**Keywords:** atrioventricular nodal reentrant, tachycardia, slow pathway

In this issue, Hirata et al. describe a retrospective multicenter study conducted to determine the reasons for recurrence of atrioventricular nodal reentrant tachycardia (AVNRT).^1^ Catheter ablation is the first‐line treatment for patients with symptomatic AVNRT, and, for quite some time, disappearance of 1:1 slow‐pathway conduction has been considered the optimal endpoint of successful treatment. Emergence of a junctional rhythm during radiofrequency (RF) energy delivery has also been considered a sensitive endpoint marker of procedural success. However, recent studies have shown residual slow pathway conduction after targeted slow pathway ablation not to be a factor influencing the recurrence of AVNRT.^2^ A recent multicenter study showed emergence of a junctional rhythm to be a sensitive but not specific marker of procedural success.^3^ Furthermore, residual dual AV nodal physiology is not a predictor of recurrence. The most reliable marker of success is noninduction of the arrhythmia when isoproterenol is administered after ablation has induced a junctional rhythm. Despite the increased sophistication of clinical endpoints, the recently reported AVNRT recurrence rate is 2.1%–3.9%.^3^ Although some patients suffering recurrence may have undergone a second session, there have been no detailed studies of patients requiring re‐treatment for AVNRT recurrence.

Hirata et al. studied 46 cases of recurrent AVNRT treated by a second ablation procedure. The 46 cases represented 1.3% of a total 3663 cases in which an initial slow pathway modification procedure had been performed. Specifically, the types of AVNRT, sites of successful ablation during the first and second sessions, treatment endpoints, and procedural data were examined in detail. The recurrent AVNRT was of the same conduction pattern as the AVNRT treated initially in 84% of patients. The site of successful ablation for the recurrent AVNRT was within the right inferior extension (RIE) of the AV node in 85% of patients, even though the initial procedure also targeted the RIE. In addition, approximately 15% of the patients with recurrent AVNRT required ablation within the coronary sinus or within the left inferior extension (LIE) on the intraatrial septum. The Hirata et al. study stands as unique and yielded novel findings, as it analyzes in detail, case by case, previously unaddressed questions regarding AVNRT recurrence.

Given the need for the creation of high‐quality ablation lesions, a possible reason for the recurrence of AVNRT may be unstable contact between the catheter tip and the target tissue during RF energy delivery. Factors contributing to such instability could include increased respiratory variability and body movements due to discomfort during the RF energy delivery and sometimes a prominent Eustachian ridge preventing placement of the ablation catheter on the atrial septum. The fact that the site for successful ablation of the recurrent AVNRT was within the RIE or even higher in Koch's triangle in 85% of the patients, despite the RIE having been targeted during the first session, suggests that insufficient lesion formation during the first session may have been involved. Although proactive use of a deflectable sheath in cases of challenging anatomy can help ensure the creation of durable lesions, new treatment endpoints need to be identified as well.

Estner et al. found recurrence of AVNRT to be more common in relatively young patients.^4^ Notably, patients included in the Hirata et al. study who suffered recurrence also tended to be relatively young, with a mean age of 53 years. Although quite sophisticated electroanatomical mapping (EAM) systems have become available in recent years, the risk of AV block with RF‐based slow pathway ablation is still not zero. From a safety perspective, RF energy delivery to the high Koch's triangle region in the first session in relatively young AVNRT patients is a matter of serious concern. It may be necessary to investigate whether relatively young patients would benefit from cryoablation high within the triangle of Koch.^5^ It is also noteworthy that most recurrences of the atypical form of AVNRT required additional treatment in the LIE region by transseptal approach. In cases of the atypical form of AVNRT, a radical cure is generally achieved during initial treatment when practitioners undertake an active left atrial approach.

The Hirata et al. study was limited in two ways. First, EAM and coronary sinus angiography may have been used simultaneously in only a few cases, which casts doubt on the reliability of comparison between the first‐ and second‐session ablation sites. Second, although most recurrent cases required reablation in the high RIE region, it cannot be ruled out that mapping and ablation from the left atrial septum may have been effective in most cases of recurrence. We must remain mindful of the fact that delivery of RF energy high within the triangle of Koch is associated with a high risk of AV block and therefore exercise caution in our initial approach to AVNRT.

Catheter ablation of AVNRT has been associated with a certain number of recurrences, despite the recently refined endpoints having been met during acutely successful procedures. Hirata and colleagues deserve praise for collecting many cases via an open social networking site and for successfully uncovering specific factors explaining recurrence after the index ablation procedure for AVNRT.

## CONFLICT OF INTEREST STATEMENT

Authors declare no conflict of interests for this article.

## References

[1] Hirata S , Kaneko Y , Tamura S , Mori H , Nishiuchi S , Tokuda M , et al. Recurrent episodes of atrioventricular nodal reentrant tachycardia: sites of ablation success, ablation endpoint, and primary culprits for recurrence. J Arrhythm. 2024;1–8. 10.1002/joa3.13060 PMC11199834 38939776

[2] Chrispin J , Misra S , Marine JE , Rickard J , Barth A , Kolandaivelu A , et al. Current management and clinical outcomes for catheter ablation of atrioventricular nodal re‐entrant tachycardia. Europace. 2018;20(4):e51–e59. 28541507 10.1093/europace/eux110

[3] Katritsis DG , Zografos T , Siontis KC , Giannopoulos G , Muthalaly RG , Liu Q , et al. Endpoints for successful slow pathway catheter ablation in typical and atypical atrioventricular nodal re‐entrant tachycardia: a contemporary. Multicenter Study JACC Clin Electrophysiol. 2019;5(1):113–119. 30678775 10.1016/j.jacep.2018.09.012

[4] Estner HL , Ndrepepa G , Dong J , Deisenhofer I , Schreieck J , Schneider M , et al. Acute and long‐term results of slow pathway ablation in patients with atrioventricular nodal reentrant tachycardia—an analysis of the predictive factors for arrhythmia recurrence. Pacing Clin Electrophysiol. 2005;28(2):102–110. 15679639 10.1111/j.1540-8159.2005.09364.x

[5] Fukuda R , Nakahara S , Wakamatsu Y , Hori Y , Nishiyama N , Sato H , et al. Cryofreezing for slow‐pathway modification in patients with slow‐fast AVNRT: efficacy, safety, and electroanatomical relation between sites of transient AV block and sites of successful cryoablation. J Cardiovasc Electrophysiol. 2021;32(12):3135–3142. 34582058 10.1111/jce.15257

